# B-LBConA: a medical entity disambiguation model based on Bio-LinkBERT and context-aware mechanism

**DOI:** 10.1186/s12859-023-05209-z

**Published:** 2023-03-16

**Authors:** Siyu Yang, Peiliang Zhang, Chao Che, Zhaoqian Zhong

**Affiliations:** 1 Key Laboratory of Advanced Design and Intelligent Computing, Ministry of Education, Dalian University, 116622 Dalian, China; 2 School of Computer Science and Artificial Intelligence, Wuhan University of Technology, 430070 Wuhan, China

**Keywords:** Medical entity disambiguation, Candidate ranking, Bio-LinkBERT, Cross-attention, ELMo

## Abstract

**Background:**

The main task of medical entity disambiguation is to link mentions, such as diseases, drugs, or complications, to standard entities in the target knowledge base. To our knowledge, models based on Bidirectional Encoder Representations from Transformers (BERT) have achieved good results in this task. Unfortunately, these models only consider text in the current document, fail to capture dependencies with other documents, and lack sufficient mining of hidden information in contextual texts.

**Results:**

We propose B-LBConA, which is based on Bio-LinkBERT and context-aware mechanism. Specifically, B-LBConA first utilizes Bio-LinkBERT, which is capable of learning cross-document dependencies, to obtain embedding representations of mentions and candidate entities. Then, cross-attention is used to capture the interaction information of mention-to-entity and entity-to-mention. Finally, B-LBConA incorporates disambiguation clues about the relevance between the mention context and candidate entities via the context-aware mechanism.

**Conclusions:**

Experiment results on three publicly available datasets, NCBI, ADR and ShARe/CLEF, show that B-LBConA achieves a signifcantly more accurate performance compared with existing models.

## Introduction

In recent years, with the development of medical technology, the volume of medical texts and medical knowledge bases have grown rapidly. It is critical to leverage the wealth of knowledge contained in these records to provide high-quality information to facilitate clinical decision-making [1]. However, many different medical concepts may have very similar mentions, and failure to disambiguate them will lead to a misinterpretation of the entire context, which will pose a great risk to healthcare-related decisions [2]. Therefore, medical entity disambiguation is key to properly utilizing such knowledge bases. Medical entity disambiguation is the task of linking a mention in a medical text to its corresponding entity in a medical knowledge base. Because the same medical entity may have more than one name, the text representation of the entity can vary due to the problems of synonyms, abbreviations, and colloquial terms. For example, “copper toxicosis” is also written as “ct”. Linking the mention “ct” to its corresponding entity ”copper toxicosis” is an instance of medical entity disambiguation. Medical entity disambiguation has a wide range of applications in research, such as biomedical question and answer [3], diagnosis and medication decision-making, predictive modeling [4], health analysis, information retrieval, and information extraction [5].

Based on deep learning methods [6], researchers have proposed some medical entity disambiguation models. For example, medical entity disambiguation has been transformed into an entity ranking problem using convolutional neural networks (CNNs) [7]. Recently, the introduction of BERT [8] has improved the performance of many natural language processing (NLP) tasks, including in the medical field [9, 10]. Medical entity disambiguation methods based on BERT models have achieved state-of-the-art results on many benchmark medical datasets [11]. However, the traditional entity disambiguation models based on BERT (such as PubMedBERT [12]) only model the current single document. Although word embedding offers contextual knowledge, it cannot capture the dependencies and rich knowledge among documents, nor can it perform multi-hop inference. Meanwhile, medical entity disambiguation has a non-linkability (NIL) problem, in which some of the medical mentions lack corresponding entities in the knowledge base. The above challenges will significantly increase the difficulty of medical entity disambiguation and may affect the ultimate value of the medical knowledge bases. Improving the performance and scalability of the method has important practical significance for medical entity disambiguation [13].

In this study, we propose a model based on Bio-LinkBERT [14] and context-aware mechanism-B-LBConA, where Bio-LinkBERT encodes mentions and entities by capturing the dependencies among documents, the cross-attention mechanism models the interaction information between mentions and entities, and ELMo encodes the context to obtain the rich disambiguation knowledge implicit in the context. Our main contributions are summarized as follows.

- Encoding mentions and entities using Bio-LinkBERT while adding character-level information to overcome the out-of-vocabulary problem.
- Modeling the relationships between mentions and entities through the cross-attention mechanism, and making full use of the interaction information between them.
- Encoding the context of mentions using ELMo, which captures lexical information, and computing the context score using a self-attention mechanism to obtain contextual cues about disambiguation.
- Showing that the model proposed in this paper outperforms existing models, including the traditional BERT-based model, through experiments on three publicly available datasets.

The rest of the article is organized as follows. Section “Related work” discusses related work on medical entity disambiguation. Section “Methodology” explains our approach and details the general structure of each module. Section “Experiments” presents an experimental validation of the proposed approach and provides an in-depth analysis of the results. Finally, Section “Conclusion” summarizes our conclusions and delineates directions for further work.

## Related work

In traditional entity disambiguation tasks, a mention needs to be accurately linked to a real entity in a common knowledge base that provides various types of information (such as entity name, entity description, entity attributes, or entity type). However, medical knowledge bases have little available information besides entity name. Therefore, although some models perform well in traditional entity disambiguation tasks, it is difficult to apply these models to professional fields that cannot provide extensive knowledge.

### Rule-based entity disambiguation methods

Early studies of medical entity disambiguation used manually defined rules to simulate text coherence between mentions and entities. The disambiguation task was typically performed by specifying some order or weight combination of these rules to calculate string similarities between the mentions and entities. Kang et al. [6] proposed an NLP module containing five rules to improve the regularity of medical texts. Souza et al. [15] used ten rules of different priorities to measure the similarities between mentions and entities and obtained desirable experimental results on the National Center for Biotechnology Information (NCBI) dataset.

Rule-based methods usually have a very high accuracy rate because when defining rules manually, we know the correct entity and always adopt the rule that tends more towards the correct entity. However, these methods have the disadvantage of very low recall, which means that the correct entity is rarely present in the candidate set.

### Machine learning-based entity disambiguation methods

To avoid manual rules, machine learning methods automatically learn the similarities between mentions and entities [16]. DNorm modeled mentions and entities using a spatial vector model and evaluated their similarities via a similarity matrix. UWM [17] performed entity disambiguation by learning the edit distances between variations of medical mentions in UMLS for diseases, whereas TaggerOne [18] used semi-Markov models, and other methods used feature-based approaches. All of the above methods have achieved good results on the NCBI dataset.

The machine learning based methods have higher recall than the rule-based methods, but they cannot distinguish similar words using semantic information [19] and they require the use of complex feature engineering for computation in order to achieve higher accuracy rate.

### Deep learning-based entity disambiguation methods

Zhu et al. [20] proposed a model that performed entity disambiguation using semantic information of mentions and entities. Vashishth et al. [21] used type information to improve entity disambiguation. Li et al. [7] introduced entity disambiguation architectures with pre-trained word embeddings for CNNs. The above approaches only allow for independent representation of each word [19], and the models do not generalize well to related words.

Shahbazi et al. [22] and Broscheit [23] proposed entity disambiguation models for contextual word embeddings based on ELMo and BERT. These models used the contextual word embeddings of words around a mention to predict the target entity. Recently, Ji et al. [11] fine-tuned the BERT model, turning the medical entity disambiguation into a sentence pair classification task, and achieved better results on medical entity disambiguation datasets. Based on the BERT model, Peng et al. [24] proposed BlueBERT, which was initialized with BERT and further trained on biomedical corpora of PubMed abstracted and clinical notes. Rohanian et al. [25] proposed BioTinyBERT, which has fewer word vector dimensions, hidden layers, and FFN layers than BERT. Although BioTinyBERT is lighter and has faster inference speed, it cannot fully capture the rich semantic information in the transformer. Liu et al. [26] introduced SAPBERT, which uses the metric learning objective function to self-align the representation space of biomedical entities. Sung et al. [27] introduced BioSyn, which uses synonym marginalization to maximize the probability of all synonym representations in candidates. However, the existing BERT-based approaches do not capture the relationships among documents and are not efficient in practice [13].

Other works have used entity textual information, such as entity descriptions, to generate entity representations. Logeswaran et al. [28] introduced the entity linking dataset in Zero-shot, with more focus on entity descriptions. Yao et al. [29] addressed remote modeling in entity descriptions by repeating location embeddings. However, as stated earlier, there is no information beyond entity name available in medical domain. In addition, the BERT-based model proposed by Logeswaran et al. [28] cannot fully capture the evidence of consistency between the mention and the target entity due to the limitation of BERT input length [30]. To address the above problems, we propose the B-LBConA model.

## Methodology

In this section, we will describe the key modules that make up the B-LBConA model and how they process input.

### Task Definition

Given a set of mention phrases (mentions with context) from a medical text document containing N mentions $\{{{M}_{1}}, {{M}_{2}}, { }\ldots, { }{{M}_{N}}\}$, a knowledge base containing M entities $\{{{E}_{1}} {, }{{E}_{2}}, { }\ldots, { }{{E}_{M}}\}$, and a training set that has correctly linked all mentions to entities, our aim is to link each mention in the test set to the correct entity in the knowledge base. We assume that there is no available information in the knowledge base other than the entity name. If there is no entity corresponding to the current mention in the knowledge base, it will be linked to NIL, indicating that the mention cannot be linked.

### Model Architecture

**Fig. 1:**
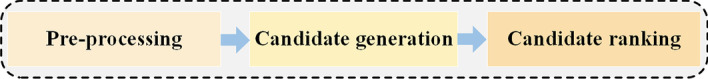
The overview of the proposed B-LBConA model

At a higher level, the B-LBConA model is divided into three modules: (1) data pre-processing, (2) candidate generation, and (3) candidate ranking. The model architecture is shown in Fig. 1.

*Pre-processing*: All mentions in the mention phrases and entity names in the knowledge base are pre-processed to unify the format for subsequent operations.

*Candidate generation*: For each mention, a candidate entity set with k candidate entities $\{{{C}_{1}} {, }{{C}_{2}}, { }\ldots, { }{{C}_{k}}\}$ is generated from the knowledge base.

*Candidate ranking*: Each candidate entity in the candidate entity set is scored by the candidate ranking module, and the candidate entity with the highest score is finally output as the target entity.

#### Pre-processing

Owing to the strong professionalism of the data, the unprocessed raw data may be very chaotic and have an incomplete structure, so we first pre-process the data to avoid unpredictable influence on the following work. The pre-processing methods are extended abbreviations, entity segmentation, number conversion and other processing.

#### Candidate generation

Owing to the particularity of the medical field, a mention may involve a large number of entities, but there is no available alias table. Therefore, we use the candidate generation module to obtain the candidate entity set $\{{{C}_{1}} {, }{{C}_{2}}, { }..., { }{{C}_{k}}\}$ of mention M so as to control the number of candidate entities. This module is crucial for the performance of the medical entity disambiguation model. In addition, the entity disambiguation model ultimately generates results from the candidate set, so we need to recall as many candidate entities as possible to ensure that the target entity matched to the mention is in the candidate set. To achieve this goal, we construct the candidate set from two aspects: exact and fuzzy matching, and similarity calculation.

*Exact and Fuzzy Matching* We select candidate entities based on entity names that exactly match all the letters with the mention or share multiple common characters with the mention. In addition, we also consider information about other mention phrases. Specifically, if the current mention is an abbreviation or substring of a mention in another mention phrase, we merge the candidates of the original mention and the extended mention. For example, the mention ”eye movement abnormalities” contains the mention ”abnormalities” as a substring, so we treat ”eye movement abnormalities” as an extended form of ”abnormalities” and add its candidates to the candidate set of ”abnormalities”.

*Similarity Calculation* The Levenshtein ratio (*LevRatio*) and cosine similarity are used to calculate the similarity between the mention and the candidates, and then the top k candidates with the highest scores are finally selected as candidates. Since entities may have multiple names, we calculate the similarity between a mention and all names of entities and take the maximum score as the score of mention *M* and entity *E*. Here, *M* and *E* are split into tokens: $M=\{{{m}_{1}}, { }{{m}_{2}}, { }\ldots, { }{{m}_{|a|}}\}$, $E=\{{{e}_{1}}, { }{{e}_{2}}, { }\ldots, { }{{e}_{|b|}}\}$. *LevRatio* is calculated as

1 $$\begin{aligned} \begin{aligned}(b) LevRatio=\frac{(|a|+|b|)-ldist}{|a|+|b|}, \end{aligned} \end{aligned}$$

where *ldist* indicates the class edit distance. Its value reflects the similarity of the string, and the top 100 entity names with the highest scores are selected.

Considering the word order problem, we calculate the aligned cosine similarity by simultaneously calculating the similarity of the mention token to the entity name token and the similarity of the entity name token to the mention token.

2 $$\begin{aligned} \begin{aligned}(b) Align{\text {Cos}}({{m}_{i}},E)&={{\max }_{{{e}_{j}}\in E}}\cos ({{m}_{i}},{{e}_{j}}) \end{aligned} \end{aligned}$$

3 $$\begin{aligned} \begin{aligned}(b) Align{\text {Cos}}({{e}_{j}},M)&={{\max }_{{{m}_{i}}\in M}}\cos ({{e}_{j}},{{m}_{i}}) \end{aligned} \end{aligned}$$

Finally, the similarity scores of mention and candidate names are calculated as the average of aligned cosine similarity.

4 $$\begin{aligned} \begin{aligned}(b) Sim(M,C)=\frac{\sum \limits _{i=1}^{|a|}{Align{\text {Cos}}({{m}_{i}},E)+\sum \limits _{j=1}^{|b|}{Align{\text {Cos}}({{e}_{j}},M)}}}{|a|+|b|} \end{aligned} \end{aligned}$$

We create ${{C}_{m}}=\{<i{{d}_{1}}, { }{{C}_{1}}, { }scor{{e}_{1}}>, { }..., { }<i{{d}_{k}}, { }{{C}_{k}}, { }scor{{e}_{k}}>\}$ for each mention m, where $i{{d}_{i}}$ is the candidate entity number, ${{C}_{i}}$ is the candidate entity name, and $scor{{e}_{i}}$ is the candidate entity similarity score. If there is a candidate entity with $score=1$, it means that this candidate is the target entity, and other candidates with $score<1$ can be deleted to improve the efficiency of the model. Next, we use the candidate ranking module on the candidate set to output the final disambiguation results.

#### Candidate ranking

**Fig. 2:**
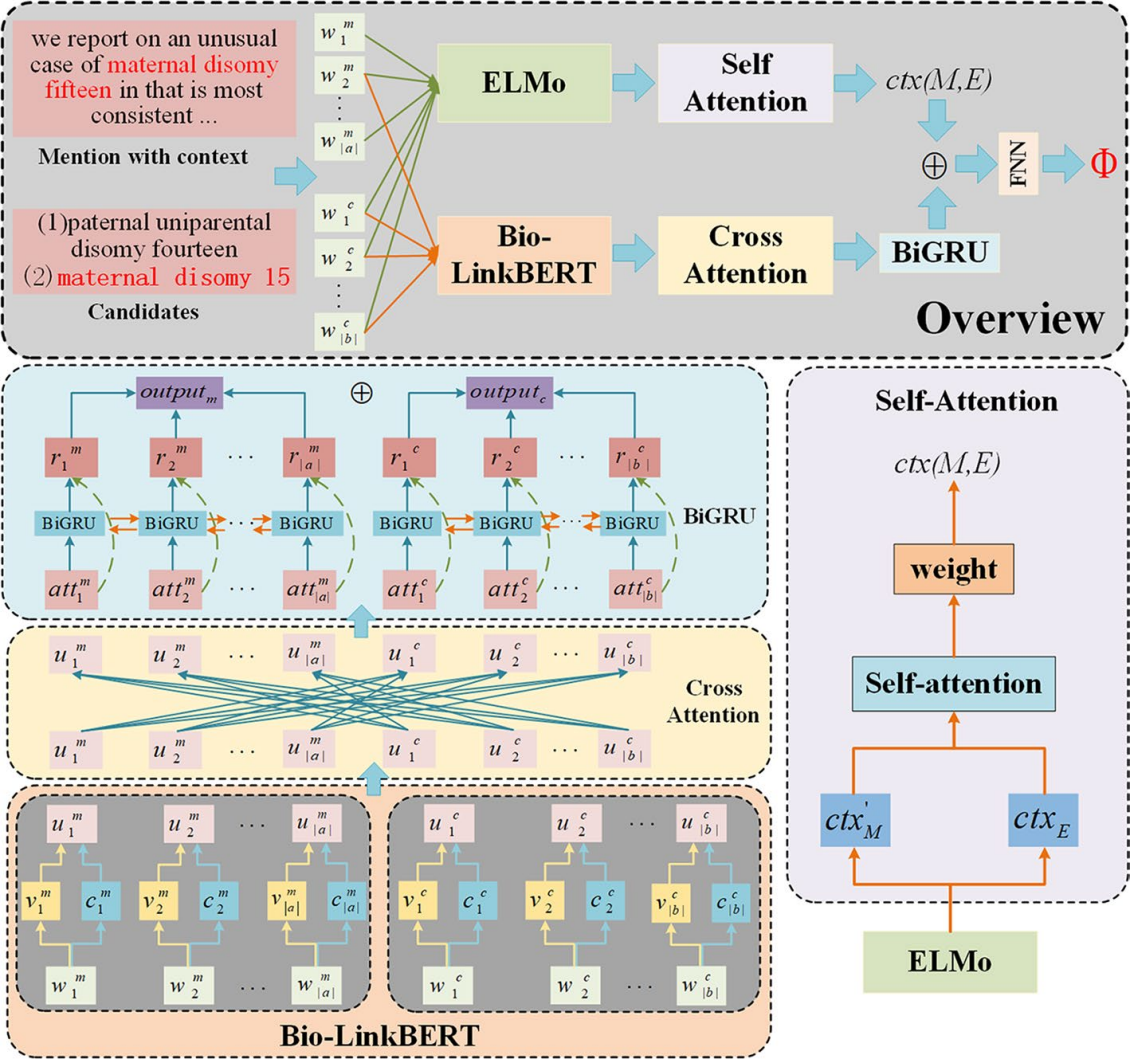
The architecture of the candidate ranking module, which takes the mention with context and entity candidates as inputs

Given a mention M and its set of candidate entities, the candidate ranking module calculates the scores of mention-candidate pairs and returns the highest scored candidate entity. The overall architecture of the candidate ranking module proposed in this paper is shown in Fig. 2, and in this section, we describe this candidate ranking module in detail. It mainly consists of an embedding layer, a cross-attention layer, a bidirectional GRU (Bi GRU) coding layer, an ELMo contextual coding layer, and an output layer. The candidate ranking module performs the following steps:

1. Mentions and candidate entities are converted into word vectors using Bio-LinkBERT, and the word vectors are linked with character-level features of each word obtained using bidirectional long-short term memory (Bi LSTM).
2. The cross-attention layer is used to capture the interaction between mentions and entities.
3. The vectors are sent to the Bi GRU layer for encoding to obtain the final representations of mentions and candidate entities.
4. A context score is calculated by self-attention to provide clues about which candidate entity to select.
5. A two-layer fully connected neural network is used to calculate the final score.

*Exact Matching* At the candidate generation phase, there is a special case where the candidate entity can completely match the mention with $score=1$. Such a mention can be linked directly to the target entity in the knowledge base and does not need to be computed in the candidate ranking module. In contrast, for entities with $score<1$ in the candidate set, the results need to be output using the candidate ranking module.

*Embedding Layer* The first layer of the candidate ranking module is the embedding layer, which concatenates the word embedding with the character embedding. In the first step, the mention token $\{w_{i}^{m}\}_{i=1}^{|a|}$ and the candidate entity token $\{w_{j}^{c}\}_{j=1}^{|b|}$ are represented using Bio-LinkBERT to obtain word embeddings $\{v_{i}^{m}\}_{i=1}^{|a|}$ and $\{v_{j}^{c}\}_{j=1}^{|b|}$. However, not all words appear in the vocabulary, so we use Bi LSTM to capture character-level features to overcome the problem of out-of-vocabulary: the Bi LSTM is run on the character sequence of each word of the mention and candidate entities to obtain the character embeddings $\{c_{i}^{m}\}_{i=1}^{|a|}$ and $\{c_{j}^{c}\}_{j=1}^{|b}$, and then the character embeddings are concatenated with the word embeddings. The final word representations $\{u_{i}^{m}\}_{i=1}^{|a|}$ and $\{u_{j}^{c}\}_{j=1}^{|b|}$ are obtained with word-level and character-level information.

*Cross-Attention Layer* In this layer, we take the word representations of the mention and candidate entities generated by the embedding layer as inputs and compute their interactions through the cross-attention module so that we can learn the relationships between text features to obtain more accurate results. As proposed in Seo et al. [31], we use a bidirectional attention mechanism: from mention to candidate and from candidate to mention. The two attentions are obtained from a shared similarity matrix $S \in {{ {\mathbb {R}}}^{m \times n}}$, which is computed from $\{u_{i}^{m}\}_{i=1}^{|a|}$ and $\{u_{j}^{c}\}_{j=1}^{|b|}$. The meaning of the elements ${{s}_{ij}}$ in the matrix is the similarity between the token *i* of the mention and the token *j* of the entity. As shown in Eq. 5, ${{W}_{a}}$ is a trainable weight vector and $\odot$ is a dot product.

5 $$\begin{aligned} \begin{aligned}(b) {{s}_{ij}}=W_{_{a}}^{\text {T}}\cdot [u_{i}^{m};u_{j}^{c};u_{i}^{m}\odot u_{j}^{c}]. \end{aligned} \end{aligned}$$

We can use *S* to obtain attention in both directions. In Eq. 7, the maximum function is calculated by column.

Mention-to-candidate Attention (M2CAtt):

6 $$\begin{aligned} \begin{aligned}(b)&{{S}^{\alpha }}=\text {softmax}(row(S){)}, \\&att_{i}^{m}=u_{i}^{m}\odot {{S}^{\alpha }}. \end{aligned} \end{aligned}$$

Candidate-to-mention Attention (C2MAtt):

7 $$\begin{aligned} \begin{aligned}(b)&{{S}^{\beta }}=\text {softmax}({{\max }_{col}}(S) {)}, \\&att_{j}^{c}=u_{j}^{c}\odot {{S}^{\beta }}. \end{aligned} \end{aligned}$$

*Bi GRU Encoding Layer* To obtain word representations containing more information, we encode the representations of the mention and candidate entities that passed through the cross-attention layer using a Bi GRU encoder to obtain $r_{i}^{m}$ and $r_{j}^{c}$:

8 $$\begin{aligned} \begin{aligned}(b)&\overrightarrow{r_{i}^{m}}=\overrightarrow{GRU}(\overrightarrow{r_{i-1}^{m}},att_{i}^{m}) { },\overleftarrow{r_{i}^{m}}=\overleftarrow{GRU}(\overleftarrow{r_{i+1}^{m}},att_{i}^{m}), \\&\overrightarrow{r_{j}^{c}}=\overrightarrow{GRU}(\overrightarrow{r_{j-1}^{c}},att_{j}^{c}) { },\overleftarrow{r_{j}^{c}}=\overleftarrow{GRU}(\overleftarrow{r_{j+1}^{c}},att_{j}^{c}), \\&r_{i}^{m}=[\overrightarrow{r_{i}^{m}};\overleftarrow{r_{i}^{m}}], { }r_{j}^{c}=[\overrightarrow{r_{j}^{c}};\overleftarrow{r_{j}^{c}}]. \\ \end{aligned} \end{aligned}$$

The GRU is a recurrent neural network capable of capturing sequential order information. GRU can only encode in one direction, so we use a Bi GRU network consisting of a forward GRU and a backward GRU. The Bi GRU concatenates the two representations obtained from sequential and reverse computations to obtain the output. Finally, the representations of the mention and candidate entities are concatenated to obtain *output*.

*Contextual Coding Layer* The context can provide disambiguation cues. In this layer, we evaluate the relevance of the mention context to the candidate entities by calculating the context score. We first encode the candidate entities and the mention context using the ELMo model with two Bi LSTM layers to obtain the candidate entities representation $ct{x}_{E}$ and the mention context representation $ctx_{M}^{\prime }$. To select important keywords and ignore the effect of noise, we use a self-attention mechanism to assign a weight to each token in the context. Then we use the weighted sum to obtain the mention context representation $ct{x}_{M}$. We compute the context score as the dot product of $ct{x}_{M}$ and $ct{x}_{E}$:

9 $$\begin{aligned} \begin{aligned}(b) ct{{x}_{score}}(M,E)=ct{{x}_{M}}\odot ct{{x}_{E}}. \end{aligned} \end{aligned}$$

Finally, we concatenate the context score into the vector *output*:

10 $$\begin{aligned} \begin{aligned}(b) output=[output,ct{{x}_{score}}]. \end{aligned} \end{aligned}$$

*Output Layer* We use two layers of fully connected neural networks to calculate the final output:

11 $$\begin{aligned} \begin{aligned}(b)&{{\Phi }^{'}}={\text {ReLU}}({{W}_{1}}\cdot output+{{b}_{1}} {)}, \\&\Phi (M,E)=\text {sigmoid}({{W}_{2}}\cdot {{\Phi }^{'}}+{{b}_{2}}). \\ \end{aligned} \end{aligned}$$

In Eq. 11, ${{W}_{1}}$ and ${{W}_{2}}$ are the learnable weight matrices, ${{b}_{1}}$ and ${{b}_{2}}$ are the bias values. The ${\text {ReLU}}$ activation function is used in the first layer and the $\text {sigmoid}$ activation function is used in the second layer.

#### NIL problem

Owing to the incompleteness of the knowledge base, a corresponding target entity cannot be found for every mention. For such mentions, entity disambiguation models usually link them to a special null entity (NIL) and cluster these null entities. We use a traditional threshold approach, where if the highest ranked candidate entity scores below a predefined threshold $\tau$, the result is NIL. The threshold $\tau$ is a value learned from the training set. For datasets that do not contain the NIL problem, we set the threshold $\tau$ to 0.

### Optimization

In this study, positive samples are randomly selected in the given training set, and negative samples are selected among the candidate entities (excluding the target entity) generated in the candidate generation phase. This makes the negative samples very similar to the positive samples, forcing the model to disambiguate entities at a finer granularity. We use the hinge loss as the loss function, which is commonly used in maximum-margin algorithms and is specific to binary classification problems. The loss function of the mention M and the candidate set C is defined in Eq. 12:

12 $$\begin{aligned} \begin{aligned}(b) {\mathcal {L}}(M,C)=\max (0,\Phi (M,{{E}^{+}})-\Phi (M,{{E}^{-}})+\mu ), \end{aligned} \end{aligned}$$

where ${{E}^{+}}$ denotes positive samples, ${{E}^{-}}$ denotes negative samples, and $\mu$ is the margin hyperparameter. The purpose of the hinge loss function is to separate positive and negative sample pairs at a certain margin by optimizing the embedding space to ensure that the positive sample pairs are close enough to each other and the negative sample pairs are far enough away from each other.

## Experiments

### Datasets

In this study, the overall performance of the B-LBConA model is evaluated on three publicly available medical entity disambiguation datasets: the NCBI-disease corpus, the TAC 2017 Adverse Reaction Extraction (ADR) dataset, and the ShARe/CLEF corpus. In the following, we present some details of these three datasets.

*NCBI* This dataset consists of 793 PubMed abstracts, 693 of which are used for training and development, and 100 for testing. The disease terms in the abstracts are manually annotated and linked to the MEDIC disease tables. In this study, we use the July 6, 2012 version of MEDIC, which contains 7827 MeSH identifiers and 4004 OMIM identifiers, and includes a total of 9664 disease concepts. Mentions without a corresponding entity in MEDIC are not annotated, so all mentions in this dataset have corresponding entity identifiers and there is no NIL problem.

*ADR* This dataset consists of 200 drug labels, 101 of which are used for training and development, and 99 for testing. The ADR in each drug label is manually mapped to the MedDRA 18.1 knowledge base, which contains 23,668 concepts. From Table 1, we can calculate that 0.7% and 0.3% of the mentions in the training set and test set are unlinkable. This illustrates the challenge of NIL in medical entity disambiguation.

**Table 1:** Dataset statistics

		NCBI	ADR	ShARe/CLEF
Train set		5932	7038	5816
Test set		960	6343	5351
Refined test		206(21.4%)	1544(24.3%)	1487(2.8%)
NIL	Train set	0	47	1641
	Test set	0	18	1750
	Refined test	0	2	536
Concepts	Train set	668	1517	1034
	Test set	203	1323	942
	Refined test	140	857	879

*ShARe/CLEF* The ShARe/CLEF corpus, which was released for an open challenge, contains 298 medical reports, 199 of which are used for training and 99 for testing. The reference knowledge base used here is the SNOMED-CT subset of umls2012aa [32]. From Table 1, we can calculate that 28.2% and 32.7% of the mentions in the training set and test set are unlinkable.

After analyzing the dataset, we find that about 80% of the entities in the test set are duplicates of the entities in training set. In order to get more real results, we process the test sets according to the method proposed by Tutubalina et al. [33], making the intersection of the training set and the test set null, and obtain the refined sets without duplicate data. We also conduct experiments on the refined sets. This operation is known as zero-shot, and the zero-shot setting demonstrates how the model maps mention to invisible entities (new entities) without tagged data in the domain, reflecting the generalization ability of the model. Table 1 shows the statistical information of the datasets, including the refined set.

### Evaluation metrics

*Recall* in Eq. 13 is the evaluation metric in the candidate entity generation phase, which denotes the probability that the model predicts to be correct among all correct entities. *Recall* measures the model’s ability to recognize positive examples, and the higher the better. *Accuracy* in Eq. 14 is the evaluation metric in the candidate ranking stage, and the higher the accuracy, the better the model effect.

13 $$\begin{aligned} \begin{aligned}(b) Recall&=\frac{TP}{TP+FN}, \end{aligned} \end{aligned}$$

14 $$\begin{aligned} \begin{aligned}(b) Accuracy&=\frac{TP+TN}{ALL}. \end{aligned} \end{aligned}$$

In Eqs. 13 and 14, *TP* denotes the number of positive samples that are correctly identified, *FN* denotes the number of missing positive samples, *TN* denotes the number of negative samples that are correctly identified, and *ALL* denotes the total number of samples.

### Baselines

To verify the effectiveness of the proposed model, we compare B-LBConA with other methods proposed in recent studies on entity disambiguation:

1. BERT-based Ranking [11]: This method fine-tunes the BERT pre-training model to set medical entity disambiguation as a sentence pair classification task.
2. Edge-weight-updating NN [34]: Entity embeddings capture more accurate information about semantic similarity between matched entities by minimizing the distributions of edge weight on the Ground Truth Entity Graph and the Similarity-Based Entity Graph.
3. SciFive [35]: A T5-based model designed for biomedical literature related tasks.
4. ED-GNN [1]: The mention in the text is represented as a query graph, and an effective negative sampling method is designed to improve the disambiguation ability of the model.
5. D-C + OD-T [36]: A text-only model that encodes mentions and entities through transformers which are trained by online hard triplet mining.
6. ResCNN [37]: Uses a residual convolutional neural network for biomedical entity linking.
7. Lightweight-NN [19]: Changes between mention and entities are captured using an alignment layer with an attention mechanism.
8. KRISSBERT [38]: It uses the domain ontology to generate self-supervised mention examples on unlabeled text, sampling the examples as prototypes for each entity, and linking by mapping the test mentions to the most similar prototypes.
9. Inter- and Intra-Attention [13]: Inter- and intra-entity attention is aggregated to capture relationships between mentions and entities and among themselves.
10. G-MAP [39]: It enhances domain-specific PLMs with memory representations built from frozen generic PLMs, without losing any generic knowledge.

### Experimental setup

We implement the proposed model using Keras and train the model on a single Intel(R) Core(TM) i9-10900F CPU @ 2.80GHz, using less than 10Gb of RAM. Adam is used as the optimizer in the experiments. Other parameters are shown in Table 2.

**Table 2:** Hyperparameter settings

Hyperparameter	Value
Character embedding dimension	128
Context sentence length	100
Learning rate	0.001
Decay rate	0.05
Batch size	128
Dropout	0.1
Epochs	30
Hinge	0.1
Top k	50

### Results

#### Performance comparison

**Fig. 3:**
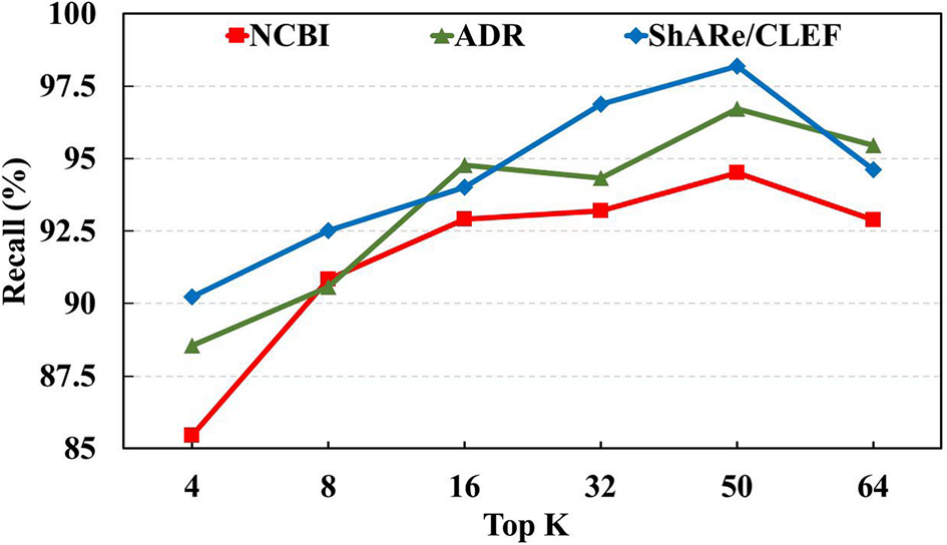
Impact of the number of top k on three datasets

In the process of generating candidate entities using the Levenshtein ratio and alignment similarity methods, we generate 50 candidate entities for each mention. The recall of correct entities on the NCBI, ADR, and ShARe/CLEF test sets is shown in Fig. 3. From the results, it can be seen that the highest recall is achieved when top k = 50, with 94.52%, 96.73%, and 98.19% recall on NCBI, ADR, and ShARe/CLEF test sets, respectively, making the candidate generation method used in this paper valid.

Table 3 shows the performance comparison results between B-LBConA and the baselines on three datasets. As the datasets are publicly available and the evaluation metrics are the same, the results of the baselines are taken from the original papers. The experimental results in Table 3 show that our model outperforms the baselines, with accuracies of 93.57%, 94.72%, and 94.23%, respectively. On the NCBI dataset, the accuracy of our model is 4.61 and 6.94 percentage points higher than the BERT-based Ranking model on official test and refined test. On the ADR dataset, the accuracy of our model is 3.07 and 4.47 percentage points higher than KRISSBERT. But on ADR’s refined test, our model is 0.16 percentage points lower than Edge-weight-updating NN, we speculate that the Edge weight updating NN optimizes the parameters of the baseline BERT model by minimizing the difference between the discrete distribution of the edge weights of the Ground Truth Entity Graph and the Similarity-Based Entity Graph. Therefore, our model can achieve better results even when facing new entities that have not appeared in the training set. On the ShARe/CLEF dataset, our model outperforms Lightweight-NN and BERT-based Ranking, suggesting that the Bio-LinkBERT model using bidirectional transformers is more effective than traditional word embedding models. Our model exceeds the ResCNN by 1.17, 0.89, and 1.44 percentage points on the three official test sets, indicating that the attention mechanism is more effective. The performance results also show that our model outperforms the current state-of-the-art model G-MAP. Lightweight-NN [19] is a lightweight entity disambiguation model. Although Lightweight-NN has fewer parameters and shorter inference time than our model, its accuracy is 1.4 percentage points lower than our model in the three datasets on average.

**Table 3:** Performance of different models

Model	NCBI		ADR		ShARe/CLEF	
	test	refined test	test	refined test	test	refined test
BERT-based Ranking [11]	88.96	67.44	93.17	79.83	91.09	80.47
Edge-weight-updating NN [34]	91.72	71.15	92.21	**80.05**	91.56	**81.45**
SciFive [35]	90.47	69.53	92.17	75.18	91.01	79.83
ED-GNN(GraphSAGE) [1]	92.44	72.36	92.03	78.25	89.46	76.39
D-C + OD-T [36]	92.25	-	-	-	90.41	-
ResCNN [37]	92.40	73.02	$\underline{93.83}$	78.96	92.79	79.13
Lightweight-NN [19]	92.56	69.65	93.07	80.34	92.73	80.78
KRISSBERT [38]	89.93	70.88	91.65	75.42	90.41	78.92
Inter- and Intra-Attention [13]	91.28	-	93.13	-	-	-
G-MAP [39]	$\underline{92.61}$	$\underline{73.75}$	93.26	79.23	$\underline{92.98}$	$\underline{81.29}$
B-LBConA (our model)	**93.57**	**74.38**	**94.72**	$\underline{79.89}$	**94.23**	80.68

The best performance on each dataset is marked in bold, and the second-best performance is marked in underline; “−” means the result is not provided

#### Ablation experiments

To demonstrate the effectiveness of each layer of the candidate ranking module in the proposed model, we construct ablation experiments with five ablation models (w/o Bio-LinkBERT, w/o character feature, w/o cross-attention, w/o Bi GRU, w/o context). The results of the ablation experiments on the three test datasets are shown in Table 4 and discussed as follows:

- *Impact of Bio-LinkBERT* When Bio-LinkBERT is not used for encoding, the performance decreases by 1.12, 0.75, and 1.23 percentage points, respectively, indicating that Bio-LinkBERT is able to obtain cross-document dependencies for better encoding of mentions and entities.
- *Impact of character features* We find that the performance after removing character features decreases by approximately 0.27, 0.32, and 0.13 percentage points on the three datasets, suggesting that character features are able to capture morphological changes at a finer granularity.
- *Impact of the cross-attention module* The performance after removing the cross-attention module decreases by 0.8, 1.14, and 1.45 percentage points, respectively, demonstrating the effectiveness of the cross-attention module in capturing information about the interaction between mention-entities.
- *Impact of Bi GRU* With the removal of Bi GRU, the accuracy decreases by 1.22, 2.98, and 2.37 percentage points.
- *Impact of context module* Removing the context module reduces the accuracy by 0.93, 0.16, and 0.89 percentage points on the three datasets, suggesting that the use of mention contexts containing rich information can further filter entities’ features. The above ablation experiments demonstrate that all layers of the candidate ranking module of our model are necessary.

**Table 4:** Ablation studies of our proposed model B-LBConA on test datasets

Model	NCBI	ADR	ShARe/CLEF
w/o Bio-LinkBERT	92.45	93.97	93.00
w/o character feature	93.30	94.40	94.10
w/o cross-attention	92.77	93.58	92.78
w/o BiGRU	92.35	91.74	91.86
w/o context	92.64	94.56	93.34
Full model	93.57	94.72	94.23

#### Comparison with other BERT-based approaches

To address the validity of the Bio-LinkBERT, we replace the Bio-LinkBERT with other BERTs: BlueBERT [24], PubMedBERT, BioDistilBERT [25], BioTinyBERT [25], BioMobileBERT [25], SapBERT [26] and BioSyn [27]. The results of the experiments are listed in Table 5, where Bio-LinkBERT shows better performance than other BERT. B-LBConA is 1.06 and 0.34 percentage points higher on the NCBI’s official test set and refined test with BioSyn(init. w/SAPBERT). On ADR dataset, the BioSyn achieved the best results due to the model’s use of synonym marginalization techniques to maximize the probability of all synonym representations in the top candidates object. On ShARe/CLEF dataset, we achieve the best and the second-best, respectively. BioDistilBERT is derived from knowledge distillation from biomedical teacher and continuous learning on Pubmed datasets. Because the teacher model with higher precision is trained in advance, then the knowledge distillation of the student model with this trained teacher model will get a higher precision model, so BioDistilBERT obtained a relatively better performance. In conclusion, the medical entity disambiguation model proposed in this paper, which mainly uses Bio-LinkBERT, has achieved better performance than other BERTs on three selected benchmark datasets.

**Table 5:** Comparison with other BERT variants

Model	Parameters	NCBI		ADR		ShARe/CLEF	
		test	refined test	test	refined test	test	refined test
BlueBERT(Fine-Tuned) [24]	110 M	88.13	69.73	92.87	79.36	90.66	74.92
PubMedBERT(Fine-Tuned) [12]	110 M	90.28	72.79	93.01	*81.96*	92.45	78.26
BioDistilBERT(Fine-Tuned) [25]	80 M	91.15	72.13	92.79	80.45	92.67	**83.29**
BioTinyBERT(Fine-Tuned) [25]	18 M	87.48	67.34	89.68	75.31	89.48	78.64
BioMobileBERT(Fine-Tuned) [25]	30 M	89.86	68.21	90.14	75.93	90.28	76.83
SAPBERT(w/o Fine-Tuned) [26]	110 M	90.02	70.41	92.37	79.52	90.89	77.47
SAPBERT(Fine-Tuned) [26]	110 M	92.34	73.25	93.42	81.64	91.37	78.59
BioSyn [27]	110 M	90.58	72.48	**95.02**	81.19	92.16	77.34
BioSyn(init. w/SAPBERT) [27]	110 M	*92.51*	*74.04*	94.65	**82.45**	*93.45*	79.85
Bio-LinkBERT (ours)	108 M	**93.57**	**74.38**	*94.72*	79.89	**94.23**	*80.68*

The bold font indicates the best performance on each dataset and the italics font indicates the second-best performance

#### Results on data sets of different sizes

**Fig. 4:**
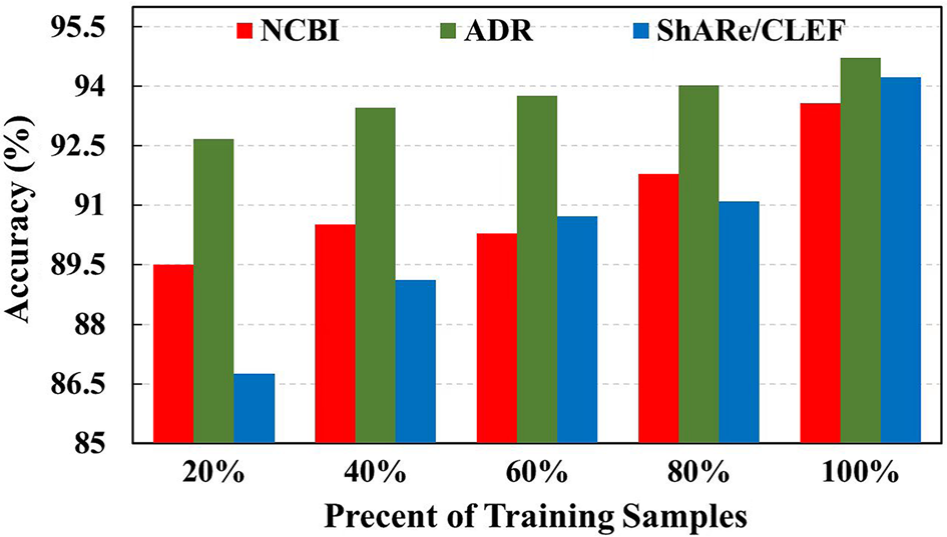
Effects of different data sizes on performance of our model

To investigate the performance of the model on different sizes of training samples, we sample the dataset twice. As shown in Fig. 4, the performance of the model improves as the number of training samples gradually increases. Even with only 20% of the training samples, the model achieves an accuracy of 89.50%, 92.67%, and 86.75% on the NCBI, ADR, and ShARe/CLEF datasets, respectively.

#### Results of different negative sampling methods

We replace our negative sampling method with other popular negative sampling methods to verify the effectiveness of our method. The experimental results are shown in Table 6. The experimental results show that our negative sampling method is the most effective and can maximize the learning ability of the model.

**Table 6:** Model performance with different negative sampling methods

Method	NCBI		ADR		ShARe/CLEF	
	test	refined test	test	refined test	test	refined test
Random Negative Sampling [40]	78.26	52.98	75.84	50.49	80.96	60.74
Popularity-biased Negative Sampling [41]	79.32	50.30	80.21	55.21	78.39	58.23
Adversarial Negative Sampling [42]	85.66	70.76	85.43	67.38	86.52	69.79
Dynamically Negative Sampling [43]	90.47	81.13	92.03	80.67	91.65	77.18
Our sampling method	**93.57**	**74.38**	**94.72**	**79.89**	**94.23**	**80.68**

The bold font indicates the best performance on each dataset

#### Error analysis

We list three representative examples of prediction error in Table 7. Based on the ground truth, the model’s prediction results are unsatisfactory for one of the following two reasons: a) one mention corresponds to multiple entities, or b) the entity name is part of the mention. In future work, we plan to improve the ability of B-LBConA to avoid these problems.

**Table 7:** Examples of prediction error

Mention	Prediction	Ground-truth
Toenail abnormalities	Toenail pitting	Toenail disorder
Colorectal carcinoma and adenomas	Colorectal carcinoma, adenomatous	Colorectal adenomas
Breast/ovarian cancer and other cancers	Breast cancer	Breast neoplasms, ovarian cancer, cancers

## Conclusion

In this study, we propose B-LBConA, a medical entity disambiguation model based on Bio-LinkBERT and context-aware mechanism. Our model uses Bio-LinkBERT to encode mentions and entities while capturing the interaction information between them using the cross-attention module; the mention context is used to obtain a context score, which measures the relevance of each candidate entity to the context to provide disambiguation cues. Extensive experiments show that our model achieves better results than the BERT-based entity disambiguation approach on three benchmark medical entity disambiguation datasets.

In future work, we plan to improve our model by (1) further improving the recall rate in the candidate generation stage, where disambiguation would be better facilitated if the target entities were more often present in the candidate entity set; (2) using additional information, such as previous knowledge, to further improve the results; and (3) designing modules that can correctly predict for the case of one mention corresponding to multiple entities.

## Data Availability

The data used in this study were obtained from the NCBI dataset(https://huggingface.co/datasets/ncbi_disease), the ADR dataset(https://bionlp.nlm.nih.gov/tac2017adversereactions/) and the ShARe/CLEF dataset(https://physionet.org/content/shareclefehealth2013/1.0/).

## Abbreviations

BERT: Bidirectional encoder representations from transformers
CNNs: Convolutional neural networks
NLP: Natural language processing
NIL: Non-linkability
NCBI: National center for biotechnology information
ADR: Adverse reaction extraction
GRU: Gated recurrent unit
Bi GRU: Bidirectional GRU
Bi LSTM: Bidirectional long-short term memory
NER: Named entity recognition
Ab3p: Biomedical text abbreviation recognition tool
LevRatio: Levenshtein ratio
CRF: Conditional random fields
